# Biophysical and Biochemical Characterization of Nascent Polypeptide-Associated Complex of *Picrophilus torridus* and Elucidation of Its Interacting Partners

**DOI:** 10.3389/fmicb.2020.00915

**Published:** 2020-05-26

**Authors:** Neelja Singhal, Archana Sharma, Shobha Kumari, Anjali Garg, Ruchica Rai, Nirpendra Singh, Manish Kumar, Manisha Goel

**Affiliations:** ^1^Department of Biophysics, University of Delhi, New Delhi, India; ^2^Regional Centre for Biotechnology, NCR-Biotech Science Cluster, Faridabad, India

**Keywords:** chaperone, circular dichroism, liquid chromatography–mass spectrometry, STRING, interactome

## Abstract

Nascent polypeptide-associated complex (NAC) is a ribosome-associated molecular chaperone which is present only in archaea and eukaryotes. The primary function of NAC is to shield the newly synthesized polypeptide chains from inappropriate interactions with the cytosolic factors. Besides that, NAC has been implicated in diverse biological functions, which suggest that it might be a multifunctional protein. An elaborate study on NAC can provide useful information on protein folding in extreme conditions in which many archaea grow. Thus, in the present study, we have studied the biophysical and the biochemical characteristics of NAC of *Picrophilus torridus*, an extreme thermoacidophilic archaeon. The study of protein–protein interactions and binding partners of a protein provides useful insights into the new/unreported roles of a protein. Thus, in this study, we have identified the binding partners of NAC in *P. torridus*. The NAC protein of *P. torridus* was cloned, expressed, and purified, and its binding partners were isolated by a pull down assay followed by identification with liquid chromatography–mass spectrometry. To the best of our knowledge, this is the first report on the biophysical and the biochemical characterization of NAC from *P. toridus* and the identification of its interacting partners.

## Introduction

Nascent polypeptide-associated complex (NAC) is a ribosome-associated molecular chaperone which is present near the peptide exit site of the translating ribosomes (Wiedmann et al., 1994). The major function associated with the NAC complex is thought to be to shield the newly synthesized polypeptide chains from inappropriate interactions with the cytosolic factors. Since most of the translating ribosomes likely associated with NAC, it was considered an abundant cellular protein, expressed in equimolar quantities relative to ribosomes (Raue et al., 2007; Hoffmann et al., 2010). Ribosome-associated chaperones vary across the three domains of life. While the prokaryotes employ the bacterial trigger factors in preventing the inappropriate folding of newly synthesized polypeptide chains (Rospert et al., 2002; Kaiser et al., 2006) in eukaryotes and archaea, this function is performed by NAC (Koplin et al., 2010; del Alamo et al., 2011). In eukaryotes, NAC is reported to be a heterodimeric protein composed of alpha- and beta-subunits, while in archaea, it was reportedly a homodimer composed of only alpha-subunits (Spreter et al., 2005).

Several recent studies indicate that, besides its primary function as a molecular chaperone, NAC is involved in many other biological functions. It has been reported to be involved in the translation and the subcellular targeting of nascent polypeptides (Wickner, 1995; Powers and Walter, 1996) and in the prevention of mistargeting of ribosome nascent chain complex (Arsenovic et al., 2012; Gamerdinger et al., 2015). Besides that, NAC has been functionally implicated in the folding of the polypeptide chain, in ribosome biogenesis, in modulating protein secretion, in regulating apoptosis, as a transcription factor, etc. (Creagh et al., 2009; Kogan and Gvozdev, 2014). Despite being implicated in diverse cellular functions, its *in vivo* role and knowledge regarding its mechanism of action are still fragmentary. An elaborate study on NAC is expected to provide further insights into protein folding in extreme conditions in which many archaea grow. Thus, in the present study, we have performed biophysical and biochemical characterization of NAC of *Picrophilus torridus* to understand its role, if any, in the thermoacidophilic adaptation of this archaeon. *P. torridus* is an interesting organism which has some unique characteristics like small genome size (1.55 Mbp), adaptability to thrive in extremely low pH (0–1) and moderately high temperatures (50–60°C), and with an intracellular pH of about 4.0 (Schleper et al., 1995).

The study of protein–protein interactions (PPIs) adds crucially to our understanding of protein function(s) and helps in the characterization of the various pathways in which cellular proteins might be involved. The identification of the binding partners of a protein provides useful insights into the new/unreported roles of a protein. Also, an analysis of the interaction network aids in identifying the mechanism(s) underlying various related biological processes. Thus, in this study we have identified the binding partners of NAC in *P. torridus*. The NAC protein of *P. torridus* was cloned, expressed, and purified, and its binding partners were isolated by a pull down assay followed by identification using mass spectrometry. To the best of our knowledge, this is the first report on the biophysical and the biochemical characterization of NAC of *P. torridus* and the identification of its interacting partners.

## Materials and Methods

### Cloning and Expression of *P. torridus* Nascent Polypeptide-Associated Chaperone

The gene encoding PtNAC (327 bases) (accession no. AE017261.1) was synthesized at a commercial facility (Genscript, United States) in pUC57 plasmid with flanking *Nhe*I and *Sal*I restriction sites. The plasmid with the insert was digested with restriction enzymes *Nhe*I and *Sal*I (30-μl reaction mixture: 3.0 μl 1X buffer, 25 μl plasmid DNA with insert, 1.0 μl *Nhe*I, and 1.0 μl *Sal*I) to obtain the free insert. The insert obtained was purified from the gel using a gel extraction kit and ligated with similarly digested pET28a(+) vector in 10 μl reaction mixture [ligation reaction in 10 μl reaction mixture: 1.0 μl ligase buffer, 1.0 μl insert, 7.5 μl digested pET28a(+) plasmid, and 0.5 μl ligase enzyme]. *Escherichia coli* DH5α cells were transformed with the pET28a(+)-PtNAC construct. Colony PCR and double-restriction digestion with enzymes *Nhe*I and *Sal*I were used for the screening of the transformants.

The pET28a(+)-PtNAC construct was isolated from the transformed *E. coli* DH5α cells and further transformed in the expression vector *E*. *coli* BL21 (DE3). The transformants were grown overnight in 50 ml Luria–Bertani broth, containing 50 μg ml^–1^ kanamycin, at 37°C and 200 rpm and inoculated (2%, v/v) again in 1 L of the same medium. The culture was kept for incubation at 37°C and 200 rpm until A_600_ of 0.6 was achieved when the protein expression was induced by adding 1 mM of isopropyl β-D-1-thiogalactopyranoside (IPTG). The induced cells were harvested after 4 h by centrifuging at 8,000 × *g*, resuspended in 70 ml of lysis buffer (1X phosphate-buffered saline, PBS; pH 7.2), and sonicated (Sonics, Vibra^TM^ cell, Connecticut, United States) intermittently for 30 min to release the intracellular protein. The cell debris was removed by centrifugation at 10,000 × *g* (30 min at 4°C). Column chromatography was performed on an AKTA Prime Plus (GE Healthcare) system. The clear supernatant was filtered through a 0.22-μm membrane (mdi: SY25KG-S) and applied to 10 ml Co^2+^-NTA beads (G Biosciences) packed in a XK16 column (Pharmacia Biotech) pre-equilibrated with 1X PBS, pH 7.2. The column was washed with five column volumes of equilibration buffer and then with wash buffer W1 (1X PBS with 20 mM imidazole), and the recombinant PtNAC was eluted using 1X PBS containing 200 mM imidazole. To remove imidazole, the eluted PtNAC protein was dialyzed in 1X PBS buffer (pH 7.2) overnight. The purity and the identity of the expressed protein was determined by sodium dodecyl sulfate-polyacrylamide gel electrophoresis (SDS-PAGE) and matrix-assisted laser desorption/ionization (MALDI-TOF) analysis, respectively.

### Chaperone Assay

The chaperone activity of PtNAC was evaluated by its capability to prevent the thermal aggregation of the substrate protein, bovine carbonic anhydrase (BCA II) (Rajaraman et al., 1996; Tomar et al., 2013). A cuvette with the sample (2 ml) was inserted in the pre-heated sample chamber (65°C) of a spectrofluorimeter containing a Peltier-controlled stirred cell, and scattering was monitored with time. The excitation and emission monochromators were set at 400 nm. BCA II (0.75 μM) heated at 65°C in 50 mM Tris-HCl buffer (pH 7.5) was used as the substrate control. Keeping the BCA II concentration (0.75 μM) constant, PtNAC was added in increasing concentration ratios, i.e., 1:0.5, 1:1, 1:2, and 1:4. For this experiment, purified NAC was dialyzed overnight in 50 mM Tris-HCl buffer (pH 7.5) and then used for estimating the chaperone activity.

### Biophysical Characterization of PtNAC Using Circular Dichroism Spectroscopy

Circular dichroism (CD) experiments were conducted in a Jasco J-815 spectropolarimeter with a Peltier-type temperature controller (Jasco CDF-426 S/15). The far-UV CD spectra of PtNAC were recorded in the wavelength range 260–190 nm using a quartz cuvette with 0.1 cm path length. The data points were collected using step resolution 0.1 nm, time constant of 2 s, and scan speed of 100 nm/min, with a spectral band width of 2.0 nm. Three accumulations per sample were recorded. The software BestSel was used for predicting the percentage of α-helices and β-sheets^1^.

### Effect of pH and Temperature

The effect of pH on the secondary structure of PtNAC was studied by incubating NAC protein (0.5 mg/ml) in a buffer of different pH, ranging from 2 to 10 (20 mM glycine HCl, pH 2.0 and 3.0; 20 mM acetate buffer, pH 4.0 and 5.0; 20 mM phosphate buffer, pH 7.0 and 8.0; and 20 mM glycine NaOH buffer, pH 9.0 and 10). The effect of temperature on the secondary structure of PtNAC was studied by incubating the protein (0.5 mg ml^–1^) for 2 min at different temperatures (20–80°C), after which changes in the structural conformation were recorded. For this, the protein was dialyzed overnight in 20 mM Tris HCl buffer (pH 7.5).

### Effect of Denaturants on the Secondary Structure of NAC

A fixed volume of PtNAC (0.5 mg ml^–1^) was incubated for 3 h with different concentrations of denaturant, guanidine hydrochloride (Gdn-HCl) (1–6 M), and urea (1–6 M), and changes in the structural conformation of PtNAC were recorded.

### Conditions of *P. torridus* Culture and Preparation of Cell Lysate

*P. torridus* (DSM 9790) was purchased from DSMZ-German Collection of Microorganisms and Cell Cultures, GmbH Leibniz Institute, Germany. Archaea were grown in 500 ml of culture medium (Arora et al., 2014) at 55°C in a shaking incubator (100 rpm). The archaeal cells were harvested in the late log growth phase by centrifuging at 10,000 × *g* for 10 min at 4°C and washed with 10 mM phosphate-buffered saline (pH 7.4). The cells were resuspended in cell lysis buffer (50 mM Tris–HCl, 10 mM MgCl_2_, 1 mM EDTA; pH 7.4) and lysed by intermittent sonication on ice for 10 min. The cell lysate was cleared by centrifuging at 14,000 × *g* for 20 min (4°C).

### Isolation of Interacting Proteins of PtNAC by Pull Down Assay

His6-PtNAC was mixed with Co^2+^-NTA-Agarose beads (the beads were washed and equilibrated with 1X PBS, pH 7.2) and incubated at 4°C for 1 h to allow it to bind with the beads. These beads were then transferred into a glass column and washed with five column volumes of the equilibration buffer, followed by washing with 1X PBS containing 50 mM imidazole, and the samples were collected to remove non-specific proteins. The beads were then washed extensively with 1X PBS to remove imidazole. Following this, 10 ml of *P. torridus* cell lysate was added to the column, which was incubated overnight at 4°C on a rocker, allowing the NAC interacting proteins to bind with the PtNAC protein associated with the beads. On the next day, the column was washed with 1X PBS buffer to remove non-interacting proteins, and the elution of the bound proteins was done in 1X PBS containing 200 mM imidazole. The samples were subjected to SDS-PAGE analysis. The interacting proteins were identified using liquid chromatography–mass spectrometry (LC–MS).

### Sample Processing for LC–MS Analysis

The methods for sample preparation for LC–MS analysis have been described previously (Kumar et al., 2018). Briefly, the proteins in the eluate were incubated with 10% trichloroacetic acid overnight at 4°C. The resulting protein precipitate was washed with 2% sodium acetate in ethanol, air-dried, and resuspended in 200 μl of 8 M urea buffer (UB), loaded in a 3-kDa filter unit (Amicon-Millipore), and centrifuged at 14,000 × *g* for 15 min. Then, 100 μl of 0.05 M iodoacetamide prepared in UB was added, mixed at 600 rpm for 1 min, again kept for incubation for 20 min, followed by centrifugation at 14,000 × *g* for 10 min, and followed by twice washing with 100 μl of UB and centrifugation at 14,000 × *g* for 15 min. Then, two washings were done with 100 μl of 0.05 M ammonium bicarbonate (ABC), and centrifugation was done at 14,000 × *g* for 10 min. After this, 40 μl of ABC and trypsin (Promega V511A) solution (enzyme/protein ratio, 1:100) was added, mixed at 600 rpm for 1 min, and kept for 16–18 h of incubation in a water bath at 37°C. The digested peptides were eluted at 14,000 × *g* for 10 min, followed by recovering the remaining uneluted peptides with another 20–30 μl of ABC. The total eluted sample was acidified with 0.1% formic acid and concentrated by speed vac to attain a final volume of 10 μl. LC–MS/MS analysis was performed in a AB SCIEX Triple TOF 5600. The MASCOT and PARAGON search engines were used for peptide identification. Proteins were selected for further study based on a 5% false-discovery rate cutoff and a minimum of two peptides per protein.

## Results

### Cloning, Expression, and Purification of NAC

**FIGURE 1:**
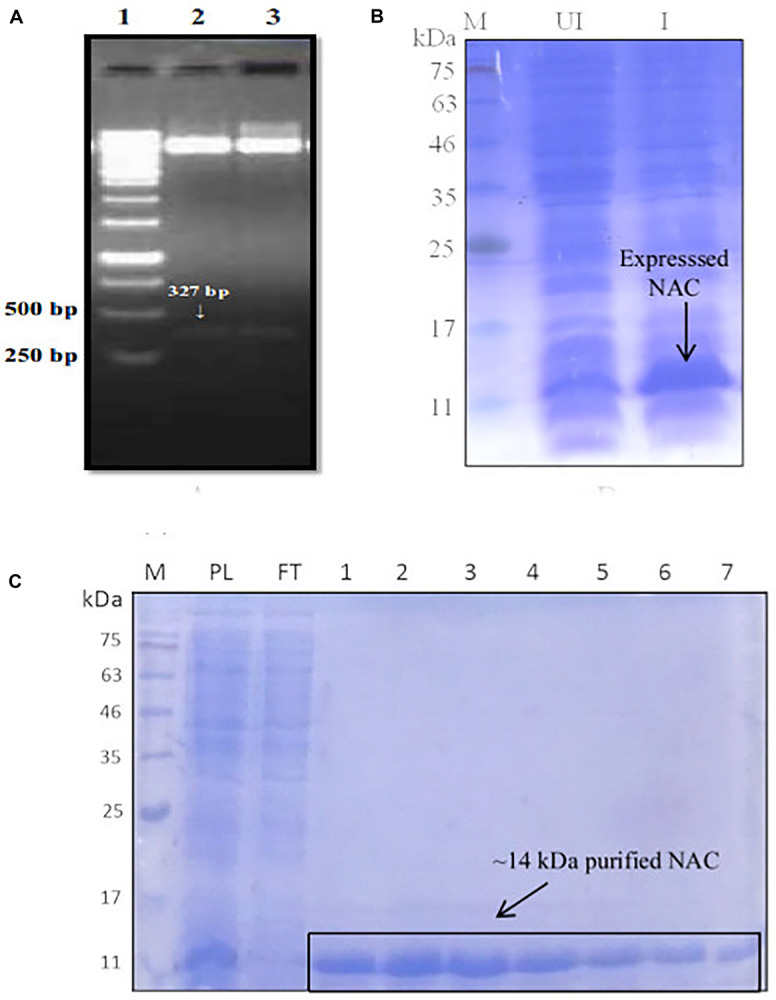
**(A)** Confirmation of positive clones of PtNAC by double-digestion method. Lanes: 1—marker (Thermo Scientific O’ GeneRuler 1-kb DNA ladder); 2, 3—double-digested pET28a(+) with the fallout. **(B)** Expression of PtNAC in *E. coli* BL21(DE3). Lanes: 1—SDS-PAGE marker, 2—uninduced: total cellular extract from *E. coli* BL21(DE3) before isopropyl β-D-1-thiogalactopyranoside (IPTG) induction, and 3—induced: total cellular extract after IPTG induction shown by arrow. **(C)** Sodium dodecyl sulfate-polyacrylamide gel electrophoresis (SDS-PAGE) analysis of recombinant NAC purified in pET28a(+) systems (samples were resolved on 15% polyacrylamide gel and stained with Coomassie Brilliant Blue R-250). Lanes: M—SDS protein marker, PL—preload, FT—flow through, 1–7 purified recombinant nascent polypeptide-associated complex eluents obtained from Co^2+^-NTA metal affinity column using imidazole (200 mM). BlueRAY prestained protein marker was used as the SDS-PAGE marker.

The positive clones encoding PtNAC were confirmed by double-digestion method (Figure 1A). The protein was found to be overexpressed in *E. coli* BL21 (DE3) (Figure 1B). The protein was purified using Co^2+^-NTA His6-affinity chromatography, followed by size exclusion chromatography on a HiPrepTM S-200 HR column (GE Healthcare). The molecular mass of the recombinant NAC protein after electrophoresis on 15% SDS-PAGE was observed to be ∼14 kDa (Figure 1C). The MALDI-TOF analysis further confirmed that the recombinant, overexpressed protein was the NAC protein of *P. torridus*. The MALDI-TOF analysis revealed two peaks corresponding to the molecular weights of 14.5 and 29.0 kDa, suggesting that the PtNAC protein purified by affinity chromatography was a mixture of dimer and monomer population (Supplementary Figure S1). However, only a single peak was observed in the elution on the gel filtration chromatography, which corresponds to the dimer population when compared to the protein standards run on this column in our laboratory (Supplementary Figure S2).

### Chaperone Activity

**FIGURE 2:**
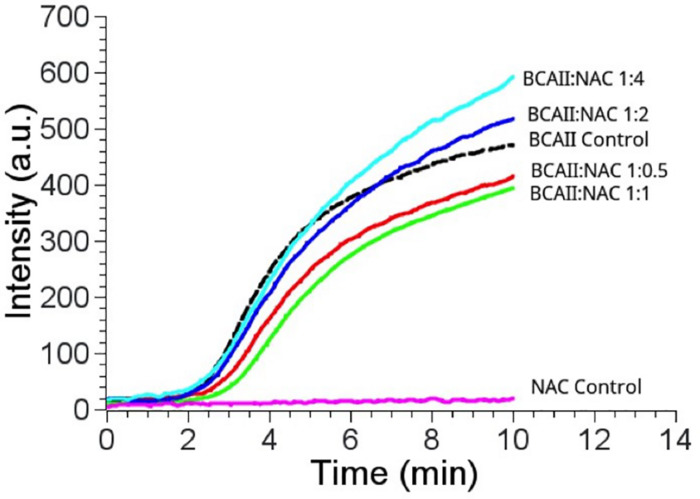
Chaperone activity of PtNAC/BCAII control is substrate control (black); BCAII/NAC in different ratios; 1:0.5 (red), 1:1 (green), 1:2 (blue), and 1:4 (cyan). The best prevention of aggregation of BCAII in the presence of NAC was observed at equimolar BCAII/NAC concentration.

There was a slight reduction in the thermal aggregation of BCAII in the presence of an equimolar concentration of NAC (BCAII/NAC, 1:1) compared to the sample containing BCAII only, suggesting that the PtNAC protein might have a chaperone-like activity *in vitro* (Figure 2). The higher concentrations of NAC were accompanied by a greater aggregation (BCAII/NAC, 1:2 and 1:4). However, the effect of the chaperone-like activity on cell viability under *in vivo* stress conditions remains to be studied.

### Effect of Temperature and pH on PtNAC Structure

**FIGURE 3:**
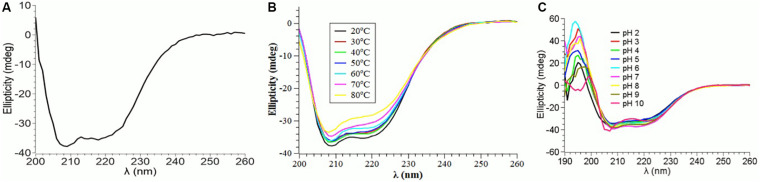
Changes in the secondary structure of PtNAC investigated using circular dichroism spectroscopy: **(A)** secondary structure estimation at pH 7.5 and 20°C, **(B)** effect of different temperatures ranging from 20 to 80°C, and **(C)** effect of different pH values ranging from 2 to 10.

The CD spectrum showed the predominance of β-sheets in the secondary structure of PtNAC at pH 7.5 and 20°C (Figure 3A). The changes in the secondary structure of PtNAC were studied at different temperatures (20–80°C), and it was observed that there was no significant change in the secondary structure with the increase in temperature up to 80°C, indicating that PtNAC was a thermostable protein (Figure 3B). The secondary structure of PtNAC was stable over a broad range of pH between 2 and 10 (Figure 3C).

### CD Spectra of the Native and Denatured PtNAC

**FIGURE 4:**
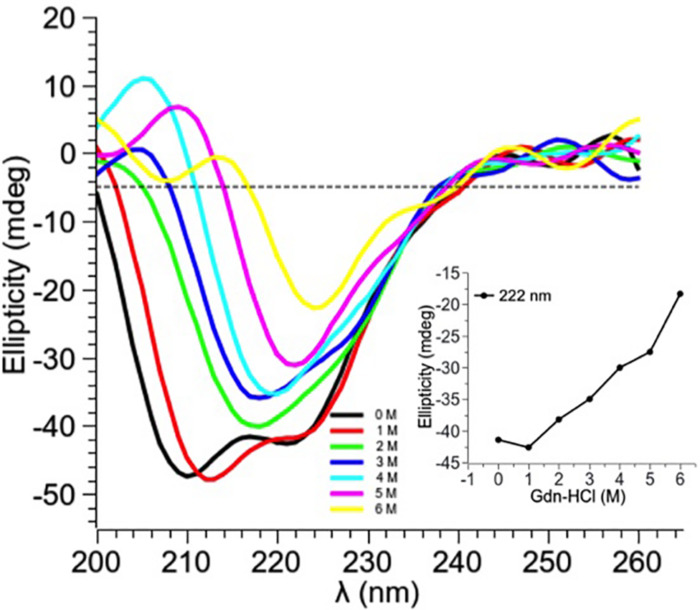
Effect of increasing concentrations of Gdn-HCl (1–6 M) on PtNAC: Inset: ellipticity at 222 nm versus Gdn-HCl concentration. On increasing Gdn-HCl concentration from 1 to 2 M, a gradual change in the secondary structure was observed. A decrease in the negative ellipticity and a right shift in the spectral wavelength were observed with the progressive increase in Gdn-HCl concentration up to 6 M.

**FIGURE 5:**
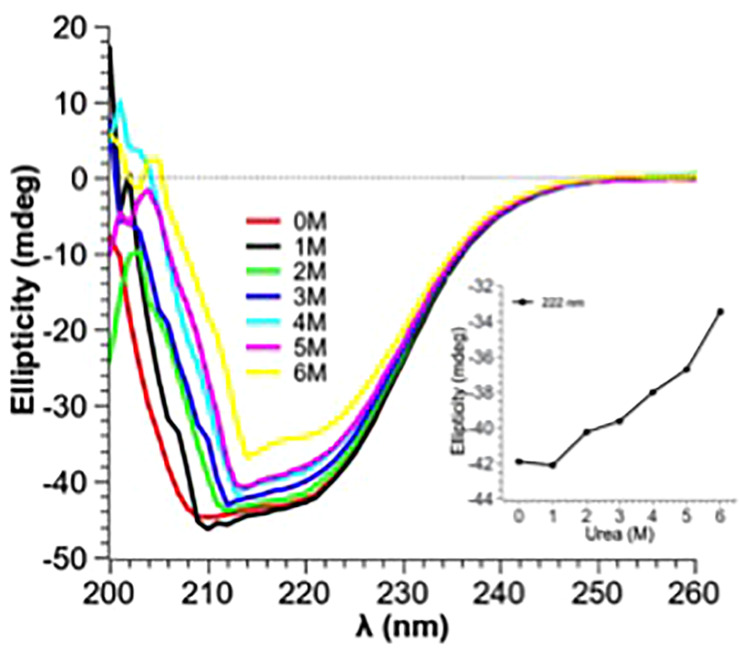
Effect of increasing concentrations of urea (1–6 M) on PtNAC: Inset: ellipticity at 222 nm versus urea concentration. A gradual change in the secondary structure was observed when the concentration of urea was increased from 1 to 6 M.

Guanidine hydrochloride (Gdn-HCl), a strong chaotrophic agent, was used to determine the effect of denaturants on the secondary structure of PtNAC. BestSel analysis predicted the presence of 21.9% ɑ-helices and 28.7% antiparallel β-sheet in the native protein (Supplementary Figure S3). However, in the presence of 6 M Gdn-HCl, the ɑ-helices content decreased significantly to 12.2%, while the antiparallel β-sheet content showed a smaller decrease to 24.9%. A progressive shift in the spectral wavelength and a decrease in negative ellipticity were observed with an increase in the concentration of Gdn-HCl up to 6 M (Figure 4, the change in secondary structure at 222 nm is shown in the inset). However, when PtNAC was incubated with increasing concentrations of urea, the decrease in negative ellipticity and the shift in spectral wavelength were not very significant (Figure 5, the change in secondary structure at 222 nm is shown in the inset).

### PtNAC-Binding Proteins

**FIGURE 6:**
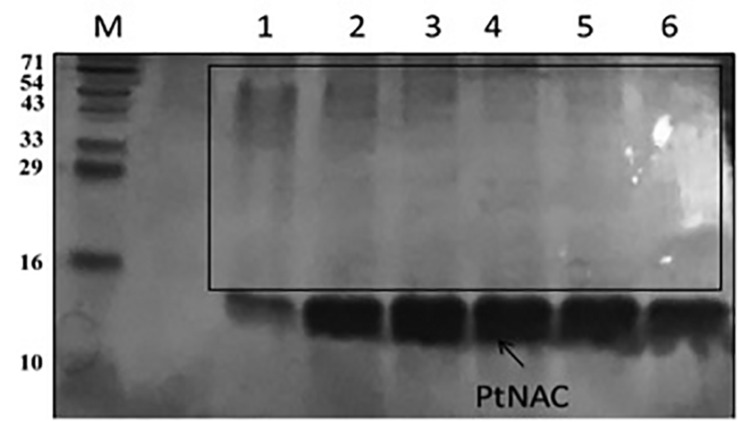
Sodium dodecyl sulfate-polyacrylamide gel electrophoresis protein elution profile of PtNAC with 200 mM imidazole after binding with *P. torridus* cell lysate: lane M—three-color prestained protein ladder from PUReGeNe; lanes 1–6 show the eluents with interacting proteins (boxed).

Protein interactions play an important role in cellular organization, and information about the functional partners of a protein can help in understanding its role in biological processes. Thus, an attempt was made to identify the interacting protein partners of PtNAC. PtNAC-binding proteins were isolated by pull down assay, separated on an SDS-PAGE (Figure 6) and identified by LC–MS. The LC–MS analysis identified 19 proteins as the binding partners of PtNAC (Table 1). These were elongation factor 1-alpha (Q6L202), protein secretion chaperonin CsaA (Q6L208), glutaredoxin (Q6L248)-related protein, thermosome subunit (Q6KZS2), 50S ribosomal protein L12 (Q6L1X7), thermosome subunit (Q6L132), hypothetical aldo-keto reductase (Q6L0H6), succinyl-CoA synthetase beta chain (Q6L0B4), DNA-binding protein (Q6L048), Rieske iron–sulfur protein (Q6KZY4), diacylglycerol-glycerol-3-phosphate 3-phosphatidyltransferase (Q6KZV0), glutamate dehydrogenase (Q6KZF2), pyruvate ferredoxin oxidoreductase, alpha chain (Q6KZA7), translation initiation factor 5A (Q6L150), serine/threonine protein kinase (Q6L2G5), homoserine dehydrogenase (Q6KZ50), and uncharacterized proteins (Q6L1Y4, Q6L168, and Q6L0Y6).

**TABLE 1:** Details of PtNAC interacting proteins identified using the pull down assay.

S. No.	Gene accession number	Protein accession number	Protein name
1	PTO0415	Q6L202	Elongation factor 1-alpha
2	PTO0409	Q6L208	Protein secretion chaperonin CsaA
3	PTO0369	Q6L248	Glutaredoxin related protein
4	PTO1195	Q6KZS2	Thermosome subunit
5	PTO0433	Q6L1Y4	Uncharacterized protein
6	PTO0440	Q6L1X7	50S ribosomal protein L12
7	PTO0699	Q6L168	Uncharacterized protein
8	PTO0735	Q6L132	Thermosome subunit
9	PTO0941	Q6L0H6	Hypothetical aldo-keto reductase
10	PTO1003	Q6L0B4	Succinyl-CoA synthetase beta chain
11	PTO1069	Q6L048	DNA-binding protein
12	PTO1133	Q6KZY4	Rieske iron–sulfur
13	PTO1167	Q6KZV0	CDP-diacylglycerol-glycerol-3-phosphate 3-phosphatidyltransferase
14	PTO1315	Q6KZF2	Glutamate dehydrogenase
15	PTO1360	Q6KZA7	Pyruvate ferredoxin oxidoreductase, alpha chain
16	PTO0717	Q6L150	Translation initiation factor 5A
17	PTO0781	Q6L0Y6	Uncharacterized protein
18	PTO0252	Q6L2G5	Serine/threonine protein kinase
19	PTO1417	Q6KZ50	Homoserine dehydrogenase OS

## Discussion

The present study aimed to unravel the biophysical and the biochemical characteristics of PtNAC and discern its binding partners. The homologs of PtNAC have been reported in all archaeal species, except *Nanarchaeum equitans, Sulfolobus solfataricus* P2, *Sulfolobus islandicus* REY15A, and *Sulfolobus islandicus* M.14.25 (Rani et al., 2016). To date, the crystal structure has been reported for NAC from only one archaeal species *Methanothermobacter marburgensis*, where it was found to be a homodimer of alpha-subunits (Spreter et al., 2005). The results of the CD spectroscopy of PtNAC indicated that ß-sheet is the predominant form of secondary structure present in PtNAC, similar to that reported in the crystal structure of NAC of *M. marburgensis* (Spreter et al., 2005).

Earlier studies had proposed that the NAC protein has a chaperone-like activity as it binds to ribosomes and interacts with nascent polypeptides (Bukau et al., 2000; Hartl and Hayer-Hartl, 2002; Wegrzyn and Deuerling, 2005). Since PtNAC mildly prevented the aggregation of heat-denatured BCAII, it might also have a chaperone-like activity. However, the effect of a chaperone-like activity on cell viability under *in vivo* stress conditions remains to be studied. The CD spectroscopic studies also revealed that the secondary structure of PtNAC was stable over a wide range of temperature and pH, which fortifies its role in aiding the adaptability of this thermoacidophilic archaea in extreme growth conditions, possibly by contributing to the prevention of aggregation and helping in the proper folding of other cellular proteins. The denaturants Gdn-HCl and urea showed different effects on the secondary structure conformations of PtNAC. This might be attributed to the difference in the nature of both denaturants. Gdn-HCl is a charged denaturant, while urea is neutral by nature. Gdn-HCl, due to its charged nature, hides the favorable electrostatic interactions that might stabilize the native state of protein, affecting the protein structure, while urea specifically binds to the amide units and therefore denatures proteins by minimizing the hydrophobic effect.

To understand the participation of NAC in metabolic processes and pathways, the present study was designed to isolate the binding proteins of *P. torridus* NAC by affinity separation followed by LC–MS analysis. The results of the pull down assay indicated that the NAC of *P. torridus* interacted with a diverse set of proteins, suggesting that it might be a multifaceted protein. The Kyoto Encyclopedia of Genes and Genomes-based metabolic pathway analysis of the NAC-binding proteins revealed that many interacting protein partners of NAC were multi-functional. A functional categorization of these proteins revealed that two of them were associated with amino acid metabolism (Q6KZ50 and Q6KZF2), two with carbohydrate metabolism (Q6L0B4 and Q6KZA7), three with energy metabolism (Q6L0B4, Q6KZA7, and Q6KZF2), four with global and overview maps (Q6L0B4, Q6KZA7, Q6KZF2, and Q6KZ50), one with metabolism of other amino acids (Q6KZF2), and one with translation (Q6L1X7) (Table 2). Interestingly, four of the interacting protein partners of PtNAC were archaeal chaperones. Two of them (Q6L132 and Q6KZS2) were subunits of thermosome that belong to the family of Group II archaeal chaperonins, one was CsaA (Q6L208) and the other was a glutaredoxin-related protein (Q6L248), a member of the thioredoxin superfamily of archaeal chaperones (Table 2).

**TABLE 2:** Annotation of Kyoto Encyclopedia of Genes and Genomes pathways of the interacting protein partners of *P. torridus* NAC.

Pathway	KEGG ID	Protein accession number	Pathway maps
Arginine biosynthesis	pto00220	Q6KZF2	Amino acid metabolism
Lysine biosynthesis	pto00300	Q6KZ50	”
Cysteine and methionine metabolism	pto00270	Q6KZ50	”
Alanine aspartate and glutamate metabolism	pto00250	Q6KZF2	”
Glycine serine and threonine metabolism	pto00260	Q6KZ50	”
Biosynthesis of secondary metabolites	pto01110	Q6L0B4, Q6KZA7, Q6KZ50	Biosynthesis of other secondary metabolites
Citrate cycle	pto00020	Q6L0B4, Q6KZA7	Carbohydrate metabolism
Propanoate metabolism	pto00640	Q6L0B4	”
Glycolysis/gluconeogenesis	pto00010	Q6KZA7	”
Butanoate metabolism	pto00650	Q6KZA7	”
C5-branched dibasic acid metabolism	pto00660	Q6L0B4	”
Pyruvate metabolism	pto00620	Q6KZA7	”
Carbon fixation pathways in prokaryotes	pto00720	Q6L0B4, Q6KZA7	Energy metabolism
Nitrogen metabolism	pto00910	Q6KZF2	”
Microbial metabolism in diverse environments	pto01120	Q6L0B4, Q6KZF2, Q6KZA7, Q6KZ50	Global and overview maps
Carbon metabolism	pto01200	Q6L0B4, Q6KZF2, Q6KZA7	”
Biosynthesis of antibiotics	pto01130	Q6L0B4, Q6KZA7, Q6KZ50	”
Biosynthesis of amino acids	pto01230	Q6KZ50	”
D-Glutamine and D-glutamate metabolism	pto00471	Q6KZF2	Metabolism of other amino acids
Ribosome	pto03010	Q6L1X7	Translation

The NAC-binding partner Q6L202 was identified as elongation factor 1-alpha. Elongation factor 1-alpha promotes the GTP-dependent binding of aminoacyl-tRNA to the A-site of ribosomes during the biosynthesis of proteins. The NAC-binding protein Q6L208 was recognized as CsaA. CsaA is one of the few chaperones which are present in bacteria and archaea but is absent in eukaryotes. CsaA prevents the aggregation of unfolded proteins and helps in the translocation of proteins across the cytoplasmic membrane (Sharma et al., 2018). The binding partner Q6L248 was identified as a glutaredoxin-related protein. Glutaredoxin is a member of the thioredoxin superfamily of proteins which is crucial for maintaining a reduced intracellular redox state. It also helps in protein folding and has been shown to have a chaperone-like activity (Berndt et al., 2008; Rani et al., 2016).

The NAC-binding proteins Q6L132 and Q6KZS2 were identified as thermosome subunits. Thermosomes belong to the family of Group II chaperonins which are ubiquitously present in archaea and impart extreme thermal stability (Phipps et al., 1991; Bigotti and Clarke, 2008). The Group II chaperonins comprise up to 40% of the total cellular protein and are abundantly produced by the cells exposed to heat shock (Trent et al., 1991). The binding partner Q6L1X7 was identified as 50S ribosomal protein L12. The 50S ribosomal protein L12 binds to the 23S rRNA and is an important constituent of the secondary structure of the ribosome. The NAC-binding protein Q6L0H6 was identified as a hypothetical aldo-keto reductase. Aldo-keto reductases are a superfamily of enzymes which are involved in the reduction of aldehydes and ketones. The binding protein Q6L0B4 was identified as the beta chain of succinyl-CoA synthase. It is a mitochondrial matrix enzyme composed of two subunits, alpha and beta. Succinyl-CoA synthase is an enzyme in the Krebs cycle that converts succinyl-CoA to succinate and free coenzyme A and converts ADP or GDP to ATP or GTP, respectively (Johnson et al., 1998).

The PtNAC-binding partner Q6KZY4 was identified as Rieske iron–sulfur protein. Proteins containing Rieske-type [2Fe-2S] clusters are associated with essential functions in all the three domains of life. The Rieske proteins occur as subunits in the cytochrome bc1 and cytochrome b6f complexes of prokaryotes and eukaryotes or form components of archaeal electron transport systems (Schmidt and Shaw, 2005). The families encoding for Rieske iron–sulfur proteins are more common in bacteria and archaea than in eukaryotes. Recent studies suggest that Rieske proteins are functionally versatile like other redox proteins, and the use of multiple Rieske proteins in electron transfer reactions helps in microbial adaptation to changing environmental conditions (Schneider and Schmidt, 2005). The binding protein Q6KZF2 was identified as glutamate dehydrogenase. Glutamate dehydrogenase is one of the most widely studied enzymes for understanding the thermostability of hyperthermophiles (Vetriani et al., 1998; Vieille and Zeikus, 2001). The binding protein Q6KZA7 was identified as pyruvate ferredoxin oxidoreductase alpha chain. In enzymology, a pyruvate ferredoxin oxidoreductase/pyruvate synthase is an enzyme that catalyzes the interconversion of pyruvate and acetyl-CoA.

The PtNAC-binding protein Q6KZ50 was identified as reversed homoserine dehydrogenase. Homoserine dehydrogenase is a key enzyme in the aspartate pathway involved in the NAD (P)-dependent reduction of aspartate beta-semialdehyde into homoserine. Homoserine is an intermediate in the biosynthesis of three amino acids—threonine, methionine, and isoleucine—in plants and microorganisms. The binding protein Q6L2G5 was identified as a serine/threonine protein kinase. Serine/threonine protein kinases are involved in protein phosphorylation, one of the most important post-translational modifications that regulate almost every cellular process, including signal transduction. The binding protein Q6L150 was identified as translation initiation factor 5A. The translation initiation factor 5A promotes the formation of the first peptide bond at the onset of protein synthesis. The binding protein Q6KZV0 was identified as CDP-diacylglycerol-glycerol-3-phosphate 3-phosphatidyltransferase. The CDP-diacylglycerol-glycerol-3-phosphate 3-phosphatidyltransferase participates in the metabolism of glycerophospholipids/phosphoglycerides. Glycerophospholipids are present abundantly in the cell membranes where they serve as an anchor for proteins in cell membranes and also participate in cell signaling.

**FIGURE 7:**
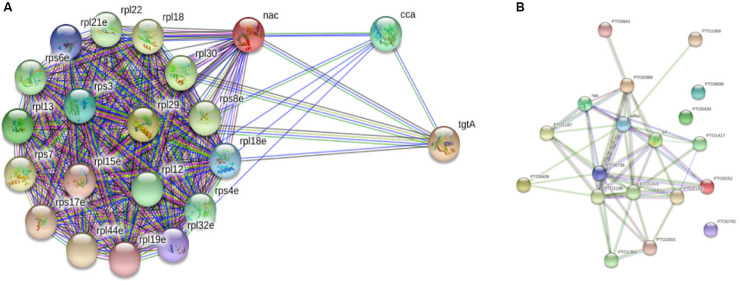
STRING database version 10.5-based protein–protein interaction analysis of PtNAC: **(A)** STRING-predicted protein binding partners of PtNAC. **(B)** Network of PtNAC and experimentally determined PtNAC-binding proteins created using STRING.

When String database version 10.5 (Szklarczyk et al., 2017) was used to discern the interacting protein partners of PtNAC, it was observed that, of the 19 interacting protein partners predicted by the STRING database, only one protein, 50S ribosomal protein L12 (rpl12), was present in the experimental interactome (Figure 7A). This is, however, not surprising because to date no experimental interactome studies have been conducted for the NAC proteins of archaea. Also, because NAC proteins are absent in prokaryotes, the NAC interaction networks in STRING might have been built on the basis of information available about eukaryotic NAC proteins. The STRING database suggests the maximum interacting partners of PtNAC to be ribosomal proteins, as predicted by other studies as well (Pech et al., 2010); however, only a single ribosomal protein was identified in our study. It is possible that the inclusion of EDTA in the binding buffers could have possibly led to the dissociation of ribosomes and affected their binding to PtNAC. Since the binding of most proteins *in vivo* is transient, the interacting partners identified in the study are also expected to be sensitive to conditions such as the buffers used for facilitating binding and subsequent separation from the column. More elaborate studies (e.g., similar pull down studies at different pH and buffer concentrations) may be required to make the predictions more robust. However, the final validation of any such predicted interaction lies in seeking the effect of such interactions on the physiology of the organism, such as growth and reproduction. However, this preliminary study does provide an opportunity to investigate the role of NAC protein in pathways or cellular functions hitherto unassigned to this protein. We therefore made an attempt to find if the interacting partners predicted in this study can be predicted to be part of a common pathway or have been shown to interact with each other in any previous studies.

Next, a network analysis of PtNAC and its interacting protein partners experimentally identified in our study was performed using String database version 10.5 (Figure 7B). Interestingly, the STRING analysis revealed a strong interaction network among the experimentally identified PtNAC-binding proteins. Sixteen of the 19 experimentally identified proteins showed mutual connections with each other on the PPI network map. The three PtNAC interacting proteins which did not integrate in the interaction network were uncharacterized proteins (protein accession/gene accession—Q6L1Y4/PTO0433, Q6L168/PTO0699, and Q6L0Y6/PTO0781). Thus, the results of the STRING analysis corroborated our experimental findings and suggested novel functional possibilities for the NAC protein.

## Conclusion

The present work is the first study of the interactome analysis of NAC of any archaeal species and, to the best of our knowledge, the first report on the biophysical and the biochemical characterization of PtNAC. Although this is a preliminary study, our results do provide an opportunity to investigate the role of NAC in pathways or cellular functions hitherto unassigned to this protein.

## Data Availability Statement

The datasets generated for this study are available on request to the corresponding author.

## Author Contributions

MG conceptualized the study. NS, AS, SK, RR, and NS worked on the methodology. AG, MK, and NS were responsible for the software. NS, AS, SK, RR, and NS validated the study. NS contributed to the formal analysis. MG  helped with the resources. NS and AS prepared the original draft. NS, AS, and MG reviewed and edited the manuscript.

## Conflict of Interest

The authors declare that the research was conducted in the absence of any commercial or financial relationships that could be construed as a potential conflict of interest.

## Footnotes

1 http://bestsel.elte.hu/
