# Supplementary material for: Biophysical and Biochemical Characterization of Nascent Polypeptide-Associated Complex of Picrophilus torridus and Elucidation of Its Interacting Partners
Source: Front Microbiol. 2020 May 26;11:915. doi: 10.3389/fmicb.2020.00915 (PMC7264160; doi:10.3389/fmicb.2020.00915)
Supplement: Supplementary file 1 [file Data_Sheet_1.docx]

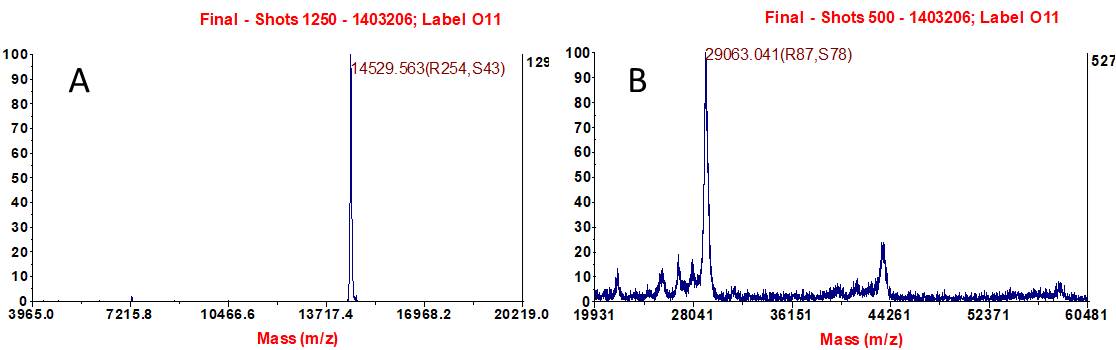


Figure S1: MALDI-TOF MS analysis- (A) Peak corresponding to molecular weight of 14.5kDa (monomer), (B) Peak corresponding to a molecular weight of 29kDa (dimer).


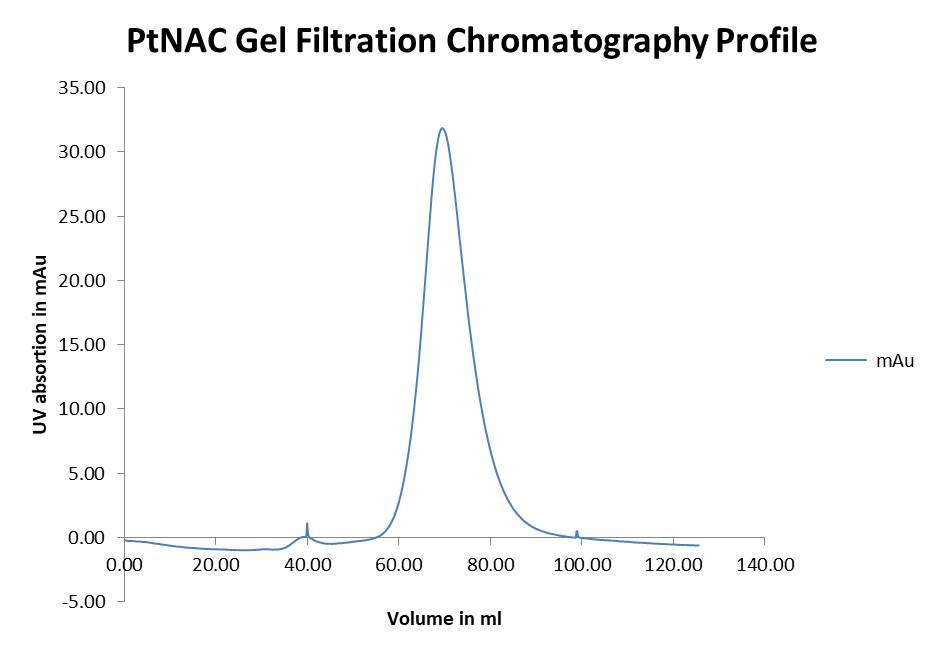


Figure S2: Gel filtration chromatography showing single peak maximum corresponding to elution volume of around 70ml suggesting dimer population.


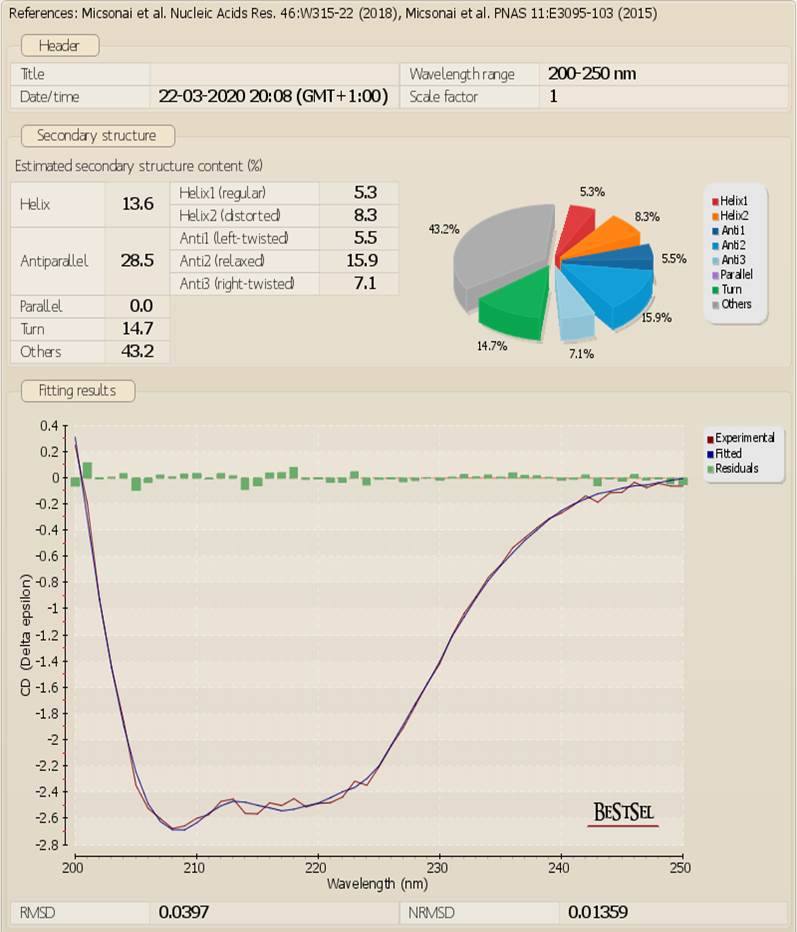


Figure S3: Bestsel CD spectroscopy analysis of PtNAC in pH 8.0 buffer showing 13.6% helix and 28.5% β-sheet.
